# Impact of different exercise modalities on neuroendocrine well-being markers among university students: a study of renalase and catecholamine responses

**DOI:** 10.3389/fphys.2025.1591132

**Published:** 2025-05-01

**Authors:** Vedat Çınar, Mehmet Fırat Bağ, Mehdi Aslan, Fidan Çınar, Alessandro Gennaro, Taner Akbulut, Gian Mario Migliaccio

**Affiliations:** ^1^ Department of Physical Education and Sport, Faculty Sport Science, Fırat University, Elazig, Türkiye; ^2^ School of Physical Education and Sports, Siirt University, Siirt, Türkiye; ^3^ Department of Psychology and Health Sciences, Pegaso Telematic University, Naples, Italy; ^4^ Department of Coaching Education, Faculty Sport Science, Fırat University, Elazig, Türkiye; ^5^ Department of Human Sciences and Promotion of the Quality of Life, San Raffaele Rome Open University, Rome, Italy

**Keywords:** exercise modalities, catecholamines, renalase, exercise, wellbeing

## Abstract

Catecholamines (epinephrine, norepinephrine, dopamine) and renalase are among the key biomolecules that regulate stress responses during exercise and support physiological adaptation. However, the effects of different exercise types on these biomolecules remain unclear. This study aims to compare the effects of aerobic, anaerobic, and strength exercises on epinephrine, norepinephrine, dopamine, and renalase levels.Materials and Methods: This study was conducted using a pre-test post-test controlled experimental research design. A total of 80 healthy male participants aged 18–22 years were included and randomly assigned into four groups: control (C), aerobic exercise (A), anaerobic exercise (An), and strength training (Sa). The exercise groups followed specific training protocols for 8 weeks, 3 days per week, at the same time of the day under standardized environmental conditions. Venous blood samples were taken before and after the exercise program, and epinephrine, norepinephrine, dopamine, and renalase levels were analyzed using the ELISA method. Results: Significant increases in epinephrine, dopamine, and renalase levels were observed depending on the exercise type (p < 0.01), while norepinephrine levels showed a significant decrease only in the aerobic exercise group (p < 0.05). Epinephrine levels increased in the aerobic (36.96%), anaerobic (35.42%), and strength training (27.45%) groups, while norepinephrine levels decreased only in the aerobic exercise group (6.38%). Dopamine levels increased in all exercise groups, with the highest change observed in the anaerobic exercise group (38.34%). Renalase levels increased in all exercise groups (p < 0.01), with the highest increase recorded in the anaerobic exercise group (29.42%).Conclusion: This study demonstrated that different exercise modalities induce specific neuroendocrine responses. All exercise types led to significant increases in epinephrine, dopamine, and renalase levels, with the most pronounced effects observed in the anaerobic exercise group. Strength training also produced similarly robust responses. Norepinephrine levels showed a significant decrease only in the aerobic exercise group, while non-significant reductions were observed in the other exercise groups. These findings indicate that exercise type distinctly modulates hormonal and enzymatic pathways involved in physiological adaptation.

## 1 Introduction

Exercise is one of the fundamental factors regulating homeostasis in the body and has a direct interaction with hormones (Hackney and Lane, 2015). The hormones released during exercise influence various physiological processes, ranging from energy metabolism to emotional balance. Specifically, catecholamines such as epinephrine, norepinephrine, and dopamine, along with biomolecules like renalase, regulate the body’s stress responses during and after exercise, supporting adaptive processes (Kruk et al., 2020). These molecules function both as neurotransmitters and hormones, forming a critical communication bridge between the central nervous system and peripheral organs (Zouhal et al., 2013a; Wybraniec et al., 2014). Therefore, the increase in hormone secretion during exercise contributes to the improvement of physical performance and the maintenance of overall health. Epinephrine (adrenaline) and norepinephrine (noradrenaline) are known as key components of the body’s response to stress, and both belong to the catecholamine group (Barth et al., 2007). Epinephrine is secreted from the adrenal medulla and rapidly enters the bloodstream through the activation of the sympathetic nervous system, facilitating energy mobilization during physical activity. Norepinephrine, on the other hand, regulates vascular contraction and blood pressure, working alongside epinephrine to coordinate the adaptive responses of the cardiovascular system. Additionally, it enhances attention and alertness in the brain, making it easier to cope with stressful situations (Grosman-Rimon et al., 2023). As a result, the increased levels of epinephrine and norepinephrine during exercise help maintain homeostasis and play a role in regulating physical performance (Athanasiou et al., 2023).

Dopamine is a key neurotransmitter primarily produced in the substantia nigra pars compacta and the ventral tegmental area of the midbrain. It plays an essential role in regulating motor control, attention, learning, motivation, and emotional responses through its projections along the nigrostriatal, mesolimbic, and mesocortical pathways (Berke, 2018; Björklund and Dunnett, 2007). Dopamine deficiency is associated with both psychological and physiological impairments, including bradykinesia, muscle rigidity, tremor (as seen in Parkinson’s disease), memory problems, fatigue, loss of motivation, difficulty concentrating, reduced capacity for pleasure, sudden depressive episodes, loss of appetite, and sleep disturbances (Berke, 2018). While its role in behavioral reinforcement and the development of addictive behaviors via the brain’s reward circuitry is well established, dopamine also significantly contributes to the regulation of exercise performance. During physical activity, dopamine levels increase, supporting neuromuscular coordination, central motor drive, and reward perception, which enhance exercise adherence and performance capacity (Meeusen and De Meirleir, 1995; Rabelo et al., 2017). Elevated dopamine levels can induce feelings of energy and euphoria, whereas low levels are linked to fatigue, hopelessness, and reduced physical and mental engagement. Importantly, regular physical activity, sufficient sleep, and a healthy diet have been shown to support dopaminergic function, serving as natural strategies to maintain optimal dopamine balance (Iversen and Iversen, 2007; Şengün et al., 2024; Bengin et al., 2024; Gür et al., 2024). Renalase is a flavin adenine dinucleotide (FAD)-dependent enzyme discovered in 2005 that plays a crucial role in the metabolism of catecholamines (epinephrine, norepinephrine, and dopamine). This enzyme, primarily produced by the kidneys, breaks down these biomolecules in circulation and exerts regulatory effects on the cardiovascular system. Renalase regulates heart rate and blood pressure, making it an important biomarker for conditions such as hypertension and heart diseases (Desir, 2009). Its functions in the cardiovascular and metabolic systems become even more prominent during exercise. By balancing the elevated catecholamine levels during physical activity, renalase helps regulate the body’s stress responses. A deficiency or dysfunction of this enzyme can lead to irregular blood pressure and impaired cardiac function (Czarkowska-Paczek et al., 2013). Exercise may play a role in preventing such adverse outcomes. However, most studies investigating the effects of exercise modalities on epinephrine, norepinephrine, dopamine, and renalase levels have focused on only a single type of exercise. The interplay between catecholamines and renalase is of particular interest in understanding the neuroendocrine adaptations to exercise. As physical activity triggers a surge in catecholamines—epinephrine, norepinephrine, and dopamine—renalase functions as a modulating agent by metabolizing these biomolecules, preventing their prolonged action and potential adverse cardiovascular effects (Desir, 2009; Luft, 2005). Furthermore, dopamine, beyond its neuromodulatory role, also serves as a substrate for renalase, indicating a feedback loop where renalase activity indirectly influences dopaminergic signaling (Desir et al., 2012). This coordinated regulation ensures that the acute benefits of catecholamine release during exercise—such as increased alertness, energy mobilization, and mood elevation—are balanced with long-term cardiovascular and metabolic stability. Therefore, examining these hormones together provides a more integrated understanding of exercise-induced physiological responses. As a result, comparisons between different exercise types remain insufficient, highlighting the need for further research in this area (Tokinoya et al., 2021; Tokinoya et al., 2020; Luo et al., 2022; Zouhal et al., 2001; Pellegrino et al., 2022). Additionally, while previous studies have examined neuroendocrine responses to exercise, research analyzing the effect sizes of these hormonal changes across different exercise modalities is lacking. Therefore, this study aims to evaluate multiple exercise types together. This approach seeks to reveal the multidimensional effects of different exercise protocols on renalase, epinephrine, norepinephrine, and dopamine, allowing for a better understanding of this enzyme’s critical role in physiological processes. In line with this, the study hypothesizes that different exercise modalities may influence epinephrine, norepinephrine, dopamine, and renalase levels to varying degrees. By analyzing the multidimensional effects of various exercise protocols on these hormones, this research aims to enhance the understanding of the critical physiological roles of these biomolecules.

## 2 Materials and methods

### 2.1 Experimental design and participants

This study was conducted using an experimental pre-test post-test research design with a total of 80 healthy male participants aged 18–22 years, who were students at Van Yüzüncü Yıl University and engaged in physical activity for health purposes. The participants had a mean age of 20.81 ± 0.87 years, a mean height of 176.23 ± 5.40 cm, and a mean body weight of 74.49 ± 9.01 kg. Based on these values, the mean body mass index (BMI) of the participants was calculated as 23.98 ± 3.25 kg/m^2^, indicating that the group fell within the normal weight range according to WHO classification.

The inclusion criteria for the study were as follows: no cardiovascular, neurological, metabolic, or endocrine diseases; no psychiatric disorders that could affect neurotransmitter metabolism; non-smoker; no use of medication or supplements; no engagement in regular exercise in the past 6 months; and voluntary consent to participate. The exclusion criteria included the presence of any health problems, smoking, use of medication or supplements, regular exercise within the past 6 months, a diagnosis of acute or chronic disease, a history of hormonal imbalance, excessive caffeine or energy drink consumption, or having experienced a significant trauma, surgery, or illness within the past 3 months. Prior to the study, the statistical power and required minimum sample size were calculated using G-power analysis. Based on an α error probability of 0.05, a power (1-β) of 0.8, an effect size of 0.88, and an alternative hypothesis (H1), it was determined that a minimum of 20 participants per group was required to detect a statistically significant difference (Zouhal et al., 2001; Cohen, 1988). Accordingly, the participants were randomly assigned into four groups: control (C), aerobic exercise (A), anaerobic exercise (An), and strength training (Sa).

**FIGURE 1:**
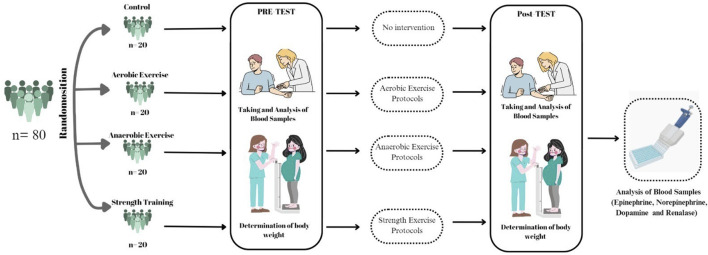
Experimental design.

Ethical approval for the study was obtained from the Non-Interventional Research Ethics Committee of Fırat University (Decision No: 348,174, dated 17/09/2019). The study was conducted in accordance with the Declaration of Helsinki and was supported by the Scientific Research Projects Coordination Unit of Fırat University under project number BSY.21.03. Participants’ height, weight, and age were recorded between 09:00 and 11:00 a.m. in a controlled environment at a constant temperature of 22°C. Venous blood samples (5 mL) were collected from the left arm using gel-containing yellow-capped tubes after 12 h of overnight fasting while participants were seated. Body mass index (BMI) values were calculated accordingly. The exercise groups (A, An, Sa) performed their exercise sessions three times per week for 8 weeks, at the same time of day and under similar conditions (Figure 1).

## 3 Training protocol

### 3.1 Control group (C)

Participants in this group did not follow any exercise program. Throughout the study, they were instructed to refrain from engaging in regular exercise or any physical activity that could affect the study outcomes. Their compliance with this requirement was closely monitored.

### 3.2 Aerobic exercise group (A)

Participants in the aerobic exercise group performed running sessions at 70%–75% of their maximum heart rate, determined using the Karvonen formula. Each training session consisted of 5 min of static warm-up and 10 min of dynamic stretching, followed by 40 min of running at a steady heart rate. Heart rates were continuously monitored using the Polar Werlink H9 Bluetooth Smart device. At the end of each session, participants performed 15 min of cool-down exercises.

### 3.3 Anaerobic exercise group (an)

Participants in the anaerobic exercise group followed a high-intensity interval training (HIIT) Tabata protocol. This protocol began with 10 min of dynamic warm-up, followed by eight sets of 20 s of high-intensity effort at 170% VO_2_ max, interspersed with 10 s of active recovery (Akbulut et al., 2022; Tabata, 2019).

### 3.4 Strength training group (Sa)

Participants in the strength training group performed resistance exercises at 65%–75% of their one-repetition maximum (1RM). The program included 10 different exercises targeting both upper and lower extremities, with each exercise performed in three sets of 8–10 repetitions. The training schedule was structured as follows: Monday—chest, biceps, and shoulders; Wednesday—back, triceps, and hamstring-focused leg exercises; and Friday—quadriceps-focused leg exercises, core, and cardiovascular exercises. Rest intervals were 30–40 s between sets and 60–90 s between exercises, ensuring a balance between muscle fatigue and recovery (Akbulut et al., 2022; Westcott, 2009) (Table 1).

**TABLE 1:** Strength training program.

Day	Exercise movement	
Monday *(Chest, Forearm, Shoulder)*	Bench Press	Preacher Curl
	Incline Dumbbell Press	Hammer Curl
	Lateral Raises	Triceps Dips
	Dumbbell Shoulder Press	Wrist Curls
Wednesday *(Back, Back Arm, Leg with Focus) Hamstring*	Deadlift	Romanian Deadlift
	Lat Pulldown	Lying Leg Curl
	Seated Row	Triceps Rope Pushdown
	Reverse Fly	Farmer’s Walk
Friday *(Leg with Focus Core, Cardio) Quadriceps*	Squat	Hanging Leg Raise
	Leg Press	Plank
	Bulgarian Split Squat	Russian Twists
	Calf Raises	Jump Rope

## 4 Data collection

### 4.1 Determination of body weight and BMI

Participants’ height was measured barefoot using a wall-mounted stadiometer (Seca, Germany) with an accuracy of ±1 mm, following the Frankfort plane, and recorded in centimeters. Body weight was measured with an electronic scale (Seca, Germany) with an accuracy of ±100 g, while participants wore light clothing (shorts and t-shirts). BMI was calculated by dividing the body weight (kg) by the square of the height (m^2^).

### 4.2 Taking and analysis of blood samples

Blood samples were collected twice—at the beginning of the study and on the final test day after completing the 8-week exercise program—from the antecubital vein using gel-containing serum separator tubes (yellow cap). After allowing the blood to clot at room temperature, the samples were centrifuged at 4,000 rpm for 5 min to obtain serum. The collected serum was then transferred into Eppendorf tubes and stored at −80°C until analysis.

Serum epinephrine, norepinephrine, dopamine, and renalase levels were analyzed using the ELISA method, following the instructions provided in the kit catalog. Absorbance measurements were performed using a BioTek ELx800 reader (BioTek Instruments, USA), while plate washing was conducted with an Auto-washer Bio-Tek ELX50 (BioTek Instruments, USA). Results for epinephrine, norepinephrine, and dopamine were expressed in pg/mL, while renalase levels were expressed in ng/mL. The measurement ranges and sensitivities of the ELISA kits were as follows: Epinephrine (Catalog No: RE10134): 31.25–2000 pg/mL, sensitivity: 9.74 pg/mL; Norepinephrine (Catalog No: RE10132): 78.13–5,000 pg/mL, sensitivity: 26.1 pg/mL; Dopamine (Catalog No: RE10135): 15.63–1,000 pg/mL, sensitivity: 4.71 ng/mL; Renalase (Catalog No: RE2517H): 7.82–500 ng/mL, sensitivity: 4.69 ng/mL.

## 5 Statistical analyses

Statistical analyses were conducted using SPSS 22 (IBM Corp, Armonk, NY). The Shapiro-Wilk test was used to determine whether the data followed a normal distribution, while Levene’s test was used to check variance homogeneity. To examine the effects of different training programs on biochemical parameters, a 2 × 2 repeated measures ANOVA was applied, evaluating the factors of time (pre-test and post-test) and group (control, aerobic, anaerobic, strength training). Based on the assumptions being met, the analysis also evaluated the time × group interaction effects. The statistical significance of within-group and between-group differences was determined using ANOVA. If a significant within-group difference was found, Bonferroni-adjusted *post hoc* paired comparisons were conducted using SPSS Syntax commands to identify which groups contributed to the pre-test and post-test differences. Statistical significance was set at p < 0.05. Effect sizes were calculated using partial eta-squared (ηp^2^) and classified according to Cohen’s (1988) criteria as small (ηp^2^ < 0.01), medium (0.01 ≤ ηp^2^ < 0.06), and large (ηp^2^ ≥ 0.14). Descriptive statistics were reported as mean ± standard deviation (SD).

## 6 Results

Table 2 presents pre- and post-test mean ± SD values for each group (C, A, An, Sa), including percentage changes and ANOVA results for Time, Group, and Time*Group interactions. F-values indicate the test statistic, p-values represent significance levels, and ES denotes effect size. Statistical significance is marked at p < 0.05, *ηp2:* Partial eta squared.

**TABLE 2:** Pre- and post-test values of biochemical parameters across exercise groups with ANOVA analysis of time, group, and Time*Group interactions.

Variables	Groups	Mean ± SD (pre)	Mean ± SD (post)	Change (%)	ANOVA (time)	ANOVA (group)	Anova (Time*Group)
Epinephrin	Control (C)	0.48 ± 0.02	0.49 ± 0.01	%2.08	F = 1,196.5 p= 0.000 *ηp2* = 0.94	F = 69.0 p = 0.000 *ηp2* = 0.73	F = 108.4 p = 0.000 *ηp2* = 0.81
	Aerobic Exercise (A)	0.46 ± 0.02	0.63 ± 0.29	%36.96			
	Anaerobic Exercise (An)	0.48 ± 0.02	0.65 ± 0.02	%35.42			
	Strength Training (Sa)	0.51 ± 0.01	0.65 ± 0.04	%27.45			
Norepinephrine	Control (C)	5.12 ± 0.01	5.11 ± 0.03	%0.02	F = 1.998 p= 0.000 *ηp2* = 0.27	F = 4.73 p = 0.004 *ηp2* = 0.015	F = 5.15 p = 0.003 *ηp2* = 0.16
	Aerobic Exercise (A)	5.17 ± 0.02	4.84 ± 0.05	%6.38			
	Anaerobic Exercise (An)	5.20 ± 0.01	5.07 ± 0.04	%2.50			
	Strength Training (Sa)	5.15 ± 0.02	4.73 ± 0.02	%8.15			
Dopamine	Control (C)	11.36 ± 1.02	11.14 ± 0.55	%1.94	F = 424.7 p= 0.000 *ηp2* = 0.84	F = 57.3 p = 0.000 *ηp2* = 0.69	F = 55.20 p = 0.000 *ηp2* = 0.68
	Aerobic Exercise (A)	11.91 ± 0.46	15.66 ± 0.86	%31.50			
	Anaerobic Exercise (An)	11.06 ±,0.67	15.30 ± 1.21	%38.34			
	Strength Training (Sa)	11.70 ±,0.94	15.83 ± 1.47	%35.30			
Renalase	Control (C)	340.0 ± 3.93	346.2 ± 3.99	%1,82	F = 6386.4 p= 0.000 *ηp2* = 0.98	F = 287.7 p = 0.000 *ηp2* = 0.091	F = 603.37 p = 0.000 *ηp2* = 0.96
	Aerobic Exercise (A)	337.3 ± 1.52	424.8 ± 8.42	%25.94			
	Anaerobic Exercise (An)	328.7 ± 4.74	425.4 ± 7.68	%29,42			
	Strength Training (Sa)	344.3 ± 8.77	438.0 ± 10.08	%27.22			

In this study, the effects of different exercise modalities on biochemical parameters were evaluated, and the changes in epinephrine, norepinephrine, dopamine, and renalase levels over time were examined. According to the results of the two-way repeated measures ANOVA, significant increases in biochemical parameters were observed in the exercise groups (p < 0.05), whereas no statistically significant changes were detected in the control group (p > 0.05).

Regarding epinephrine levels, statistically significant increases were observed in the exercise groups between the pre-test and post-test measurements (p < 0.001), while the change in the control group was not statistically significant (2.08%, p > 0.05). Epinephrine levels increased by 36.96% in the aerobic exercise group, 35.42% in the anaerobic exercise group, and 27.45% in the strength training group. The main effects of time (F = 1,196.5, p = 0.000, ηp^2^ = 0.94), group (F = 69.0, p = 0.000, ηp^2^ = 0.73), and the time*group interaction (F = 108.4, p = 0.000, ηp^2^ = 0.81) were found to be statistically significant.

For norepinephrine levels, a 6.38% decrease was observed in the aerobic exercise group, while the anaerobic exercise and strength training groups showed 2.50% and 8.15% changes, respectively. The main effects of time (F = 1.998, p = 0.000, ηp^2^ = 0.27) and the time*group interaction (F = 5.15, p = 0.003, ηp^2^ = 0.16) were statistically significant. However, the group effect was not statistically significant (F = 4.73, p = 0.004, ηp^2^ = 0.015). The change observed in the control group (0.02%) was not statistically significant (p > 0.05).

Regarding dopamine levels, the aerobic exercise group showed a 31.50% increase, the anaerobic exercise group a 38.34% increase, and the strength training group a 35.30% increase. The main effects of time (F = 424.7, p = 0.000, ηp^2^ = 0.84), group (F = 57.3, p = 0.000, ηp^2^ = 0.69), and the time*group interaction (F = 55.20, p = 0.000, ηp^2^ = 0.68) were all statistically significant. However, the 1.82% change in the control group was not statistically significant (p > 0.05).

For renalase levels, a 25.94% increase was observed in the aerobic exercise group, a 29.42% increase in the anaerobic exercise group, and a 27.22% increase in the strength training group. The main effects of time (F = 6386.4, p = 0.000, ηp^2^ = 0.98), group (F = 287.7, p = 0.000, ηp^2^ = 0.091), and the time*group interaction (F = 603.37, p = 0.000, ηp^2^ = 0.96) were statistically significant. However, the 1.94% change in the control group was not statistically significant (p > 0.05).

In conclusion, all biochemical parameters showed statistically significant increases over time in the exercise groups, while no significant changes were detected in the control group (p > 0.05). The most pronounced increases were observed in dopamine and renalase levels, with the time*group interactions demonstrating large effect sizes.

## 7 Discussion

This study examined the effects of different types of exercise on epinephrine, norepinephrine, dopamine, and renalase levels. The results showed that all exercise modalities led to a significant increase in epinephrine levels. While norepinephrine levels decreased in the aerobic exercise group, minor changes were observed in the anaerobic and strength training groups. Dopamine levels increased across all exercise groups, with the greatest change occurring in the anaerobic exercise group. Similarly, renalase levels increased in all exercise groups, with the highest increase recorded in the anaerobic exercise group. In contrast, no significant changes were detected in any of the biochemical parameters in the control group. Overall, the findings indicate that different types of exercise induce distinct biochemical responses in the neuroendocrine system.

It is well known that exercise stimulates the sympathetic nervous system, increases catecholamine secretion, and contributes to cardiovascular and metabolic adjustments (Tidgren et al., 1991). The physiological responses required for exercise adaptation have been reported to vary significantly depending on exercise intensity (high, moderate, low), duration (short, moderate, long), and type (continuous or intermittent) (Mastorakos et al., 2005). Based on the hypothesis that different exercise modalities would have distinct effects on epinephrine, norepinephrine, dopamine, and renalase levels, the findings of this study confirmed that changes in epinephrine and norepinephrine levels align with this hypothesis. Previous research has also indicated that exercise increases sympathetic nervous system activation, which is associated with a greater cardiovascular workload (Athanasiou et al., 2023). The findings of this study further demonstrate that epinephrine and norepinephrine responses vary depending on the type of exercise, reinforcing the role of exercise in cardiovascular and neuroendocrine regulation. During endurance exercise, catecholamines typically follow different time courses, with norepinephrine rising rapidly and epinephrine showing a more gradual increase (Hoffman, 2005).Additionally, individuals engaged in regular endurance training exhibit a greater epinephrine response (Zouhal et al., 2013b). In high-intensity interval exercise (HIIE), both epinephrine and norepinephrine levels increase rapidly, but this elevation generally returns to baseline within approximately 1 hour (Peake et al., 2014; Bracken et al., 2009; Kliszczewicz et al., 2018). Furthermore, 7 weeks of regular HIIE training has been shown to lead to a significant reduction in catecholamine responses, indicating that the body adapts to this stress over time (Bracken and Brooks, 2010).

Resistance exercise has been reported to cause a peak increase in epinephrine and norepinephrine levels at the end of exercise, with larger muscle group exercises eliciting more pronounced increases (Fatouros et al., 2010; Goto et al., 2008). Similarly, in short-duration dynamic exercises, catecholamine levels remain unchanged unless heart rate increases by more than 30 bpm. However, high-intensity exercise leads to a more substantial rise in epinephrine and norepinephrine levels (Christensen and Brandsborg, 1973; Bloom et al., 1976; Strobel et al., 1999; Zouhal et al., 2013a). While long-duration exercise results in a continuous increase in norepinephrine levels, short-duration and very high-intensity exercise can increase epinephrine levels by 5–20 times (Galbo et al., 1976; Koivisto et al., 1982; Kjær, 1989). Besides intensity and duration, the body region targeted during exercise has also been shown to influence catecholamine responses. For instance, upper-body exercises have been reported to induce greater catecholamine secretion than lower-body exercises. Furthermore, isometric exercises increase catecholamine secretion due to vascular compression, although this increase varies depending on the type of exercise. In dynamic exercises, norepinephrine levels show a more pronounced increase, whereas in isometric exercises, the epinephrine increase is more significant. In dynamic exercises, norepinephrine levels exhibit a more pronounced increase, whereas in isometric exercises, the increase in epinephrine levels is more significant (Watson et al., 1980; Davies et al., 1974; Zouhal et al., 2013b). When comparing the findings of this study with the existing literature, it is evident that exercise intensity, duration, and type influence catecholamine (epinephrine and norepinephrine) responses at different levels. This study provides a significant contribution to the literature by evaluating the effects of these factors on catecholamine levels within a single research framework. The results support and expand the existing knowledge on the distinct effects of different exercise modalities on the neuroendocrine system. However, it should be noted that the decrease in norepinephrine levels observed in the aerobic exercise group is not entirely consistent with previously reported findings in the literature. These discrepancies may be attributed to factors such as exercise duration, intensity, timing of measurements, and participants’ training history. Previous studies have demonstrated that as exercise intensity and duration increase, norepinephrine levels show a significant rise, with high-intensity exercise leading to greater norepinephrine release (Gökbel and Dölek, 1998). However, norepinephrine levels have also been reported to return to baseline rapidly during the post-exercise recovery period (Peronnet et al., 1981), suggesting that the timing of measurements in this study may have influenced the observed results. Additionally, it has been reported that in individuals who engage in regular exercise, the catecholamine response to a given workload diminishes over time, which may explain the adaptation in norepinephrine secretion observed in this study (Kim et al., 2015; Lovas et al., 2020). Therefore, the decrease in norepinephrine levels detected in this study may not only be attributed to the physiological effects of exercise but also to underlying mechanisms such as neural regulation, metabolic adaptations, and individual differences.

Another key variable examined in this study was dopamine, which plays a crucial role in understanding the neuroendocrine effects of exercise. Recent studies have highlighted the relationship between different exercise modalities and dopamine levels (Shimojo et al., 2019). This neurotransmitter, which is essential for reward and motivation mechanisms, has been shown to be influenced by various forms of physical activity, including aerobic exercise and high-intensity interval training (HIIT) (Arazi et al., 2017; Sacheli et al., 2019; Tyler et al., 2023; Jonasson et al., 2019). Comparative studies have indicated that both HIIT and moderate-intensity continuous training improve cardiovascular health and physical fitness, but HIIT offers greater advantages in dopamine modulation (Batacan et al., 2016; Liqiang et al., 2019). Similarly, a study conducted by Arazi et al. (2017) reported that both resistance exercise and aerobic training increased dopamine levels, although resistance exercise resulted in a higher increase, which was not statistically significant. Another study examining the effects of moderate-intensity step-aerobics, spinning, and educational game exercises on dopamine levels found that all exercise programs led to a statistically significant increase in dopamine levels. Among these, spinning exercise induced the most pronounced increase, whereas step-aerobics and educational game exercises also increased dopamine levels but to a lesser extent (Yavuz et al., 2022). Another crucial factor influencing the effect of exercise on the dopaminergic system is exercise duration. In this context, a study evaluated the effects of treadmill exercise of different durations on the expression of tyrosine hydroxylase (TH), an enzyme involved in dopamine synthesis. The study implemented 10-, 30-, and 60-min treadmill exercise protocols, and the results indicated that 30 min of exercise was the most effective duration for increasing dopamine levels. While the 10-min exercise did not produce a significant effect, the 60-min exercise resulted in a lower dopaminergic response compared to the 30-min session. These findings suggest that the effects of exercise on the dopaminergic system depend not only on exercise type and intensity but also on duration (Ji et al., 2014). Previous studies have demonstrated that aerobic exercise, HIIT, and resistance training can increase dopamine levels, with this effect varying depending on exercise type, duration, and intensity (Arazi et al., 2017; Batacan et al., 2016; Yavuz et al., 2022; Botcazou et al., 2006). Similarly, our study found that exercise modulates the dopaminergic response and that different exercise modalities and durations exert varying effects on dopamine levels. In this regard, our study not only aligns with previous research by examining the effects of exercise type, intensity, and duration on dopamine levels, but it also provides a comprehensive analysis by incorporating multiple exercise modalities and assessing their effect sizes. The results indicate that anaerobic exercise produced the greatest increase in dopamine levels, resistance training had a strong effect, while aerobic exercise resulted in a lower yet significant increase. These findings highlight the importance of carefully planning exercise type, duration, and intensity to optimize dopaminergic responses. In our study evaluating the effects of exercise on the neuroendocrine system, another key biomolecule examined was renalase. This enzyme, which plays a crucial role in catecholamine metabolism, is particularly important for maintaining cardiovascular balance. Since sympathetic activity increases with exercise, the regulation of epinephrine, norepinephrine, and dopamine levels through renalase highlights the enzyme’s role in physiological adaptation (Tokinoya et al., 2021). In this study, the effects of different exercise modalities on renalase levels were assessed and compared with findings from the existing literature. In this context, both acute and chronic exercise have been shown to significantly influence plasma renin levels (Patlar and Ünsal, 2019). Additionally, the response of the renin-angiotensin-aldosterone system (RAAS) to exercise has been reported to depend on multiple factors, including exercise intensity, duration, age, and gender (Patlar and Ünsal, 2019). However, most existing studies have focused on the effects of specific exercise types on renalase independently. For instance, Tokinoya et al. (2020) observed a significant increase in plasma renalase concentrations following a 60-min moderate-intensity treadmill exercise, classified as acute exercise. In addition to acute exercise, studies have shown that 14 weeks of chronic aerobic training also increases renalase levels (Luo et al., 2022). Similarly, a human study reported that long-term aerobic exercise increased renalase levels in individuals engaged in regular endurance training. In this study, which included amateur endurance runners, serum renalase levels were measured at different time points during a 30 km run, and it was found that exercise led to a significant increase in renalase levels (Yoshida et al., 2017). While the effects of acute and chronic aerobic exercise on renalase levels have been well documented, recent findings by Tokinoya et al. (2021) suggest that HIIT also plays a significant role in this context. Accordingly, this study adopts a comprehensive approach by evaluating the effects of 8-week anaerobic, aerobic, and strength training programs on renalase levels, presenting results that align with findings in the existing literature. While most studies in the literature have examined the effects of these exercise types separately, this study directly compares the three training models, providing a detailed analysis of the different mechanisms through which exercise influences renalase regulation.

## 8 Strengths and limitations

This study provides a significant contribution to the literature by evaluating the effects of aerobic, anaerobic, and strength training over 8 weeks within a single framework, rather than analyzing different training types separately. By directly comparing three different exercise models, this study comprehensively analyzed the distinct mechanisms and effect sizes of neuroendocrine responses. The study not only assessed the presence of hormonal changes but also evaluated their magnitude, with detailed biochemical analyses of epinephrine, norepinephrine, dopamine, and renalase levels. This comprehensive approach offers valuable scientific insights into exercise-based rehabilitation, cardiovascular health, and metabolic disease management. Additionally, the inclusion of a control group allowed for a clearer distinction between exercise-induced changes and natural physiological variations, providing a more objective evaluation of exercise’s specific effects on hormonal levels. However, several limitations should be considered. The study was conducted on healthy male individuals within a specific age range, meaning that gender and age factors were not accounted for. Moreover, the study did not examine the long-term effects of exercise, nor did it analyze other metabolic markers such as cortisol, insulin sensitivity, or lactate levels. Furthermore, the absence of performance-based measurements limits the ability to fully integrate the physiological and biochemical effects of exercise. Future research should incorporate larger sample sizes and long-term follow-ups to further elucidate the comprehensive effects of exercise on the neuroendocrine system.

## 9 Conclusion

This study examined the biochemical responses of the neuroendocrine system to different types of exercise (anaerobic, aerobic, and strength training) over an 8-week period. The findings suggest that exercise plays a crucial role in catecholamine metabolism, cardiovascular adaptation, and neuroendocrine homeostasis. Notably, changes in epinephrine, norepinephrine, dopamine, and renalase levels varied depending on the type of exercise. These findings highlight the importance of carefully planning exercise type, duration, and intensity to optimize dopaminergic and neuroendocrine responses.

## Data Availability

The original contributions presented in the study are included in the article/supplementary material, further inquiries can be directed to the corresponding author.
